# Defining Expressive Language Benchmarks for Children with Down Syndrome

**DOI:** 10.3390/brainsci12060743

**Published:** 2022-06-06

**Authors:** Angela John Thurman, Lauren Bullard, Leona Kelly, Caitlyn Wong, Vivian Nguyen, Anna J. Esbensen, Jennifer Bekins, Emily K. Schworer, Deborah J. Fidler, Lisa A. Daunhauer, Carolyn B. Mervis, C. Holley Pitts, Angela M. Becerra, Leonard Abbeduto

**Affiliations:** 1 Department of Psychiatry and Behavioral Sciences, University of California Davis Health, Sacramento, CA 95817, USA; bullard@ucdavis.edu (L.B.); lakelly@ucdavis.edu (L.K.); caiwong@ucdavis.edu (C.W.); vivng@ucdavis.edu (V.N.); ljabbeduto@ucdavis.edu (L.A.); 2 MIND Institute, University of California Davis, Sacramento, CA 95817, USA; 3 Division of Developmental and Behavioral Pediatrics, Cincinnati Children’s Hospital Medical Center, Cincinnati, OH 45229, USA; anna.esbensen@cchmc.org (A.J.E.); jennifer.bekins@cchmc.org (J.B.); emily.schworer@cchmc.org (E.K.S.); 4 Department of Pediatrics, University of Cincinnati College of Medicine, Cincinnati, OH 45267, USA; 5 Department of Human Development and Family Studies, Colorado State University Fort Collins, Fort Collins, CO 80523, USA; deborah.fidler@colostate.edu (D.J.F.); lisa.daunhauer@colostate.edu (L.A.D.); 6 Department of Psychological and Brain Sciences, University of Louisville, Louisville, KY 40292, USA; cbmervis@louisville.edu (C.B.M.); holley.pitts@louisville.edu (C.H.P.); angela.becerra@louisville.edu (A.M.B.)

**Keywords:** down syndrome, expressive language, outcome measures, early communication

## Abstract

Establishing expressive language benchmarks (ELBs) for children with Down syndrome (DS), as developed by Tager-Flusberg et al. for children with autism, is critically needed to inform the development of novel treatments, identify individualized treatment targets, and promote accurate monitoring of progress. In the present study, we assessed ELB assignments in three language domains (phonology, vocabulary, and grammar) for 53 young children with DS (CA range: 2.50–7.99 years) using standardized assessments. The participants were classified into one of four ELB levels (preverbal, first words, word combinations, and sentences) in each language domain. Associations with additional measures of language, chronological age, nonverbal cognition, and verbal short-term memory were considered. Analyses of individual ELB profiles indicated substantial variability across the three language domains, with six different patterns of variation across domains emerging. At the same time, the ELB categories were significantly associated with independent language measures and broader developmental domains. Moreover, ELB changes were observed in a small sample of children with DS reassessed 18–24 months after the initial visit. Results from the present study suggest the procedures outlined by Tager-Flusberg et al. for defining ELBs are a potentially useful tool for describing the language abilities of children with DS.

## 1. Introduction

Down syndrome (DS), with an estimated prevalence of 1 in 792 live births [1], results from an extra full or partial copy of chromosome 21 and is the most common neurogenetic syndrome associated with intellectual disability. Although a great deal of heterogeneity is observed, expressive language is an area of particular challenge for most children with DS. Indeed, language delays are observed in nearly all individuals with DS, are typically more severe than their delays in nonverbal cognition, and are often more severe than the language delays associated with other neurogenetic syndromes [2,3,4]. Language is a crucial area of focus for research and a high-priority treatment target because it plays a critical role in supporting long-term outcomes.

An expert panel formed by the National Institute on Deafness and Communication Disorders (NIDCD) outlined a framework for defining expressive language benchmarks (ELBs) that describes children’s current language level and facilitates monitoring progress over time [5]. This panel highlighted that, although language outcomes are frequently considered in descriptive and intervention research, it is difficult to compare the results across studies because of differences in the procedures used to measure language levels of the participants at study entry and over time within the study [5]. In addition, terms such as “functional speech” or “minimally verbal” are often operationalized differently across studies [5]. The panel provided guidelines for generating ELBs from commonly collected language assessment data (e.g., standardized assessments) across multiple language domains (e.g., phonology, vocabulary, and grammar) to provide a developmental framework that can facilitate comparisons of participants’ language abilities within and across studies. When using standardized assessments, the ELBs are defined using developmental-level estimates, or age-equivalent scores, based on patterns observed in children with typical development (TD) between 1 and 4 years of age [5]. Four stages of expressive language development were proposed: (1) preverbal (i.e., < 15-month developmental level); (2) first words (i.e., 15- to 23-month developmental level); (3) word combinations (i.e., 24- to 35-month developmental level); and (4) sentences (i.e., 36-month developmental level or higher). The ELBs have been used to characterize the language skills of children with autism [6,7]. However, no studies have characterized the ELBs of young children with other neurodevelopmental conditions such as DS. Because language is a high-priority intervention target for children with DS, ELBs may be useful for describing children’s current language level and monitoring progress over time. However, because children with DS demonstrate both relative strength and challenge within the language domain, the extent to which the ELB framework accounts for such asynchronies remains unknown.

### 1.1. Expressive Language Development in Down Syndrome

Expressive language can be conceptualized in terms of different interrelated components, with children learning how to produce and combine sounds and words to communicate with those around them. Thus, monitoring development across multiple domains (e.g., phonology, vocabulary, grammar) informs our understanding of a child’s overall expressive language skills.

#### 1.1.1. Phonology

Before any child can produce and combine words, they must first learn how to articulate and form the sounds. Phonological development begins well before the onset of first words, with children transitioning across multiple vocal stages before they produce identifiable speech [8]. As children learn to combine sounds into words, errors are common. For example, children with TD may omit final consonants in words (e.g., /ka/ instead of /kat/), produce an earlier developing sound in place of a later developing one (e.g., /wed/ instead of /red/), or reduce a consonant blend (e.g., /kool/ instead of /skool/) [9].

In youth with DS, multiple factors make the perception and production of speech more challenging (e.g., cognitive limitations, hearing loss, anatomical/physiological differences) [10]. As a result, phonological skills remain an area of clinical focus often into adulthood [10,11,12]. Research suggests that the sequence of vocal development is similar in infants with DS and infants with TD [10,13], but delays become more apparent as children age. Phonological errors often persist through childhood and are more variable in individuals with DS than in individuals with TD [10,14,15]. Phonological challenges may also alter how individuals with DS use spoken language. For example, Miller and Leddy [16] posited that the negative impact of reduced intelligibility also causes individuals with DS to limit their talking; this, in turn, can alter developmental trajectories and opportunities for engaging in meaningful and sustained conversations with others.

#### 1.1.2. Vocabulary

The production of first words marks a turning point in expressive language development. Children with TD produce their first words around 12 months of age [17]. The rate of vocabulary growth is slow initially and then shifts to a period of rapid growth often observed in the second year of life [18,19,20]. Most children with TD transition from first words to the production of word combinations in the second year of life [21,22,23].

Expressive vocabulary skills are often an area of strength in children with DS relative to performance in other language domains, such as expressive grammar. Nonetheless, delays relative to chronological age expectations and receptive vocabulary ability are common [4,24,25]. For example, Berglund et al. [26] found that 12% of 12–23-month-olds, 80% of 24–35-month-olds, and 90% of 36–47-month-olds had produced their first word in a survey of over 300 children with DS between 1 and 5.50 years of age. Subsequent vocabulary development also occurs more slowly in children with DS than in children with TD, with 50% of children with DS at four years of age and 25% of children with DS at five years of age demonstrating a vocabulary of fewer than 50 words [26]. Delays in expressive vocabulary persist, with modest growth continuing into early adolescence, followed by evidence of a plateau in growth around 16 years of age [27].

#### 1.1.3. Grammar

Building on phonological and vocabulary development is grammatical development, which refers to the systematic way words and morphemes are combined to express meaning. Two-word combinations often emerge between 16 and 20 months in children with TD [28]. Grammatical skills continue to develop, with children increasing their use and complexity of morphemes and sentence structures with age [29].

Expressive grammar skills are an area of particular challenge for youth with DS [4]. Specifically, children with DS have significant delays in the onset of two-word combinations, with most children not demonstrating two-word combinations until after 3.50 years of age [26]. In addition, Harris [30] found that the syntactic performance of children with DS aged 2.50–6.75 years was comparable to that of children with TD aged 1.42–2.41 years. Expressive grammar weaknesses are generally apparent relative to both nonverbal cognitive ability and receptive grammar skills [31,32,33,34]. These grammar challenges have been found to persist into adulthood [4].

### 1.2. Current Study

We currently lack a consensus on the best way to characterize progress in language development in a concise, descriptive way for individuals with DS. Moreover, because studies often vary in terms of the measures used to assess language performance, it can be challenging to compare the language levels of the participating children across research and treatment studies. The ELB procedures offer one possibility for addressing this need. In the present study, we extended the use of the ELBs proposed by Tager-Flusberg et al. [5] to describe the language profiles of young children with DS across three language domains: phonology, vocabulary, and grammar. First, we examined the range of ELBs, within and across domains, observed in a sample of children with DS ranging in age from 2.50–7.99 years at their initial visit. Due to the significant expressive language delays observed in children with DS, we expected our participants to be distributed across the full range of ELB milestones [26,35].

Second, we considered the association between ELBs and (1) other language metrics not utilized in the classification process and (2) other developmental characteristics known to relate to language ability, such as chronological age (CA), nonverbal cognitive ability, and verbal short-term memory. In order to be an effective metric of expressive language ability, the ELBs must be significantly correlated with other variables designed to measure language development or that are known to be closely linked with language development [36]. We therefore hypothesized that the ELBs would be significantly associated, across language domains, with the language validation measures and with developmental characteristics associated with language performance (e.g., age, nonverbal cognition, and verbal short-term memory).

Finally, we considered longitudinal changes in ELBs (across an 18–24-month period) in a subset of participants who completed their follow-up visit. Indeed, if ELBs are an effective metric of expressive language ability, the ELBs should be sensitive to developmental changes [36]. We therefore hypothesized that significant changes in the ELBs at the initial and longitudinal follow-up visit would be observed in our subset of participants who completed their longitudinal follow-up visit.

## 2. Materials and Methods

### 2.1. Participant Characteristics

Participants were children with DS between the ages of 2.50 and 7.99 years. Caregivers provided medical reports confirming the diagnosis of DS (e.g., karyotype). Additional eligibility criteria, based on caregiver report, were (1) no uncorrected visual or hearing impairments, (2) English as the primary language used in the home, and (3) basic motor skills needed to support completion of study procedures, such as the ability to transition in and out of sitting positions independently, reach for toys while sitting, and be independently mobile (e.g., scoot, crawl, or walk). Participants were recruited through national listservs, regional DS groups, as well as existing lab databases and were enrolled from across the United States to participate in a larger longitudinal study at one of four different sites: the University of California Davis MIND Institute, Cincinnati Children’s Hospital Medical Center, Colorado State University, and the University of Louisville. Visits were scheduled to avoid changes in medications designed to manage behavior, behavioral therapy, or educational programming (not including regular school holidays/vacations) in the four weeks before the study visit. Study procedures were approved by the Institutional Review Boards at all participating universities. Parents/caregivers provided informed consent on behalf of their children.

The present study includes data from 53 children with DS (25 female) between the ages of 2.50 and 7.99 years (M = 4.78 years, SD = 1.51) at their initial visit. Mean nonverbal cognitive ability standard score was 51.04 on the Differential Ability Scales—second edition (range = 30–77, SD = 12.60). The racial representation of the present sample was: White (83.0%), Asian (5.7%), Black/African American (1.9%), Other (3.8%), Multiracial (3.8%), and Unknown/Preferred not to answer (1.9%). In addition, 15.1% of the sample identified as Hispanic or Latino/a, and 3.8% of the sample selected Unknown/Preferred not to report ethnicity. Most children lived in a two-parent household (94.3%); two children lived in a one-parent household, and one child lived in two separate one-parent households. Household income bracket (determined using data from the Pew Research Center based on annual household income accounting for household size and geographic region) was as follows: 11.3% of the children came from lower-income households, 58.5% from middle-income households, 9.4% from middle-upper income households, 15.1% from upper-income households, and 5.7% families preferred not to report their household income.

Fourteen participants in the present study had also completed the longitudinal follow-up visit at the time of data analysis. At their initial visit, these participants were between the ages of 2.72 and 6.28 years (M = 4.81, SD = 0.97). Mean nonverbal cognitive ability standard score was 47.43 on the Differential Ability Scales—second edition (range = 30–68, SD = 10.83). The racial representation of this subset of participants was: White (85.7%), Asian (7.1%), and Unknown/Preferred not to answer (7.1%). None of the participants in this sample identified as Hispanic or Latinx and 7.1% of the sample selected Unknown/Preferred not to report ethnicity. All but one child in this subsample lived in a two-parent household. Household income bracket (determined using data from the Pew Research Center based on annual household income accounting for household size and geographic region) was as follows: 14.3% of the children came from lower income households, 64.3% from middle income households, 7.1% from middle-upper income households, and 14.3% from upper income households. The average time between the initial and the longitudinal follow-up visits for this subsample was 1.78 years (range = 1.60–2.01, SD = 0.10).

### 2.2. Measures

#### 2.2.1. Expressive Language Benchmarks

ELBs were assessed in three domains: phonology, vocabulary, and grammar. As outlined by Tager-Flusberg et al. [5], the criteria for assigning ELBs, using standardized assessment procedures, were based on age-equivalent scores reflecting the expected emergence of benchmark abilities in children with TD (i.e., preverbal: 14 months or less; first words: 15 to 23 months; word combinations: 24 to 35 months; and sentences: 36 months or greater). Multiple assessment procedures were needed to assess the full ELB range for the phonology and vocabulary domains. The overall ELB was defined as the lowest ELB level a child earned for the phonology, vocabulary, and grammar domains. The benchmarks for each domain were assigned using the minimum age-equivalent scores recommended by Tager-Flusberg et al. [5]. Specific information regarding the assessment procedures used to define each benchmark, and the scores from each test that correspond to the age-equivalent scores are provided below. See Table 1 for a summary of the ELB criteria used across language domains.

*Phonology ELB.* A two-step process was used to assess phonological skills. First, examiners inventoried the child’s production of eight consonants that emerge early in typical development (i.e., m, n, b, p, d, y, w, h) and the words referentially produced during the assessment visit (hereafter referred to as the Articulation Screening Test). Children who produced at least four consonants (i.e., a development level of 15 months) [5] and five words were administered the Goldman–Fristoe Test of Articulation-3 (GFTA-3) Sounds in Words subtest [37]. The Sounds in Words subtest evaluates articulation skills when labeling single words in individuals aged 2 to 21 years. In the present study, 62% of the sample passed the Articulation Screening Test. Because many children with DS between 2 and 7 years of age have limited vocabulary sizes, administration procedures were modified such that the examiner had the child imitate the target word.

*Vocabulary ELB*. Multiple measures were also used in the current study to assess vocabulary skills. We used the Vocabulary Checklists from the MacArthur–Bates Communicative Development Inventories [23], a parent–informant measure that assesses early communication skills. Specifically, the Vocabulary Checklists scores from two separate forms were computed: (1) the CDI: Words and Gestures (CDI-W&G), which assesses vocabulary skills across an 8- to 18-month developmental level and (2) the CDI: Words and Sentences (CDI-W&S), which assesses vocabulary skills across a 16- to 30-month developmental level. Caregivers completed the CDI-W&S form, which has embedded in it the vocabulary words from the CDI-W&G form. When completing the Vocabulary Checklist, parents were asked to indicate each word spoken, manually signed, or produced using both methods spontaneously (not in imitation or song). The child’s expressive vocabulary size reflected the total number of words produced in any form (i.e., spoken, manually signed, or both); the CDI-W&G Vocabulary checklist includes 396 words, and the CDI-W&S Vocabulary checklist includes 680 words. Performance on the Differential Ability Scales—II (DAS-II) [38] The Naming Vocabulary subscale was used to measure vocabulary performance beyond the developmental window from the CDI Vocabulary Checklist.

*Grammar ELB.* Grammar skills, across all ELBs, were assessed using the Preschool Language Scales Fifth Edition (PLS-5) [39] Expressive Communication domain. The PLS-5 is a standardized, comprehensive language assessment designed for children from birth to 7 years of age. In the present project, items that allowed consideration of parent reports did not conflict with direct observations. Although Tager-Flusberg et al. combined the Preverbal and First words stages for the grammar domains, we differentiated these ELB stages using a minimum developmental level of 15 months for the first words stage (as is done for all other language domains) in the present study [5].

Importantly, the PLS-5 assesses performance across multiple language domains (e.g., items on the PLS-5 could consider vocabulary, pragmatics, or grammar skills and are interspersed). As a result, when working with populations with variable language profiles, it is possible for a child to be assigned to a grammar ELB using the age-equivalent score recommendations but not demonstrate the skills suggested by the benchmark name (i.e., prelinguistic, first words, word combinations, or sentences). For example, a child with a PLS-5 age-equivalent score between 24 and 35 months would be assigned to the word combinations ELB even if they were still only producing first words. In the present study, follow-up analyses were used to identify the number of children demonstrating this type of inconsistency. Specifically, we compared children’s assigned grammar ELB to the language skills observed by the examiner during the PLS-5 administration, using the examiner scores on the PLS-5 items considering first-word labels (item 26), word combinations (item 29), and 4- or 5-word sentences (item 33).

#### 2.2.2. Language Validation Measures

Children’s performance on the language variables collected in the study but not used in the ELB classification process was used to examine the construct validity of the ELBs. Four variables generated from three assessment procedures were considered.

*Vineland Adaptive Behavior Scales—3rd Edition, Comprehensive Interview Form* [40]. Growth scores from the Vineland-3 Receptive Communication and Expressive Communication subdomains were utilized.

*MacArthur–Bates Communicative Development Inventories: Words and Sentences (CDI-W&S)—Grammar Complexity*. The CDI-W&S Grammar Complexity score was used in study analyses. In this section, caregivers of children who have begun to combine words review 37 pairs of sentences that differ in length and grammatical complexity and indicate which sentence most resembles how the child currently talks. The sentence complexity score reflects the number of sentence pairs for which the parent endorsed the child to use the more complex form. Children who had not yet begun to combine words received a sentence complexity score of 0.

*Differential Ability Scales—II* [38] *Verbal Comprehension.* The DAS-II Verbal Comprehension subtest ability score, which is similar to a growth score, was used in study analyses. Within this subtest, children demonstrate their comprehension of oral instructions by pointing to pictures or manipulating toys.

#### 2.2.3. Other Validation Measures (Associated Developmental Domains)

Children’s performance on other measures collected in the study that are known to be associated with language performance was also used to consider the validity of the ELBs. Two variables generated from one assessment procedure were considered.

*Differential Ability Scales—II* [38] *Picture Similarities.* The ability score (similar to a growth score) on the DAS-II Picture Similarities subtest was used in study analyses. In this subtest, children are presented with a multiple-choice picture matching task and assessed on their ability to match pictures with a common element concept (e.g., match two round objects among other shapes or match a foot to a sock among other clothing items). This subtest provides a measure of nonverbal cognitive ability, which is known to be associated with language performance [38].

*Differential Ability Scales—II* [38] *Recall of Digits—Forward.* The ability score (similar to a growth score) on the DAS-II Recall of Digits-Forward subtest was used in study analyses. In this subtest, children are presented with an untimed forward digit recall and assessed on their forward number span/auditory attention. Number strings are presented at a pace of two numbers per second. This subtest provides a measure of verbal short-term memory performance, which is known to be associated with language performance [38].

#### 2.2.4. Data Collection

All study visits were conducted at the participating research sites. It is important to note that the data for approximately 60% of our assessment visits were collected prior to the COVID-19 pandemic. For those collected during the pandemic, COVID-19-related safety measures were employed. Examiners wore transparent surgical masks (ClearMask^TM^), approved by the FDA and approved for use in the European Economic Area (i.e., CE-marked). Most of the children were not able to independently mask (88.5%) and so were mask-free during testing with parental permission.

#### 2.2.5. Analysis Plan

To address our first aim, descriptive analyses were used to assess the range of ELBs observed. To address our second aim, Spearman rank order correlations were utilized to evaluate the correlations between the ELBs across domains and the correlations between the ELBs and validation measures. Nonparametric statistics were used due to the ordinal nature of the ELBs. To address our final aim, Wilcoxon signed-rank tests were used to consider changes in ELBs across an 18- to 24-month period for the 14 children with DS who had completed their longitudinal follow-up visit; this nonparametric statistical hypothesis test was used due to the ordinal nature of the ELBs.

## 3. Results

### 3.1. Expressive Language Benchmarks

Descriptive statistics for performance on the assessments used to define ELBs are presented in Table 2. Every child was assigned to an ELB for each language domain and an overall ELB level. Due to incomplete GFTA-3 administrations or examiner error, the phonology ELB could not be determined for four participants. Because in all four cases, the participants earned a minimum of a first words phonology ELB, and this was not the lowest ELB observed, the overall ELB could be determined for all participants. 

**Figure 1:**
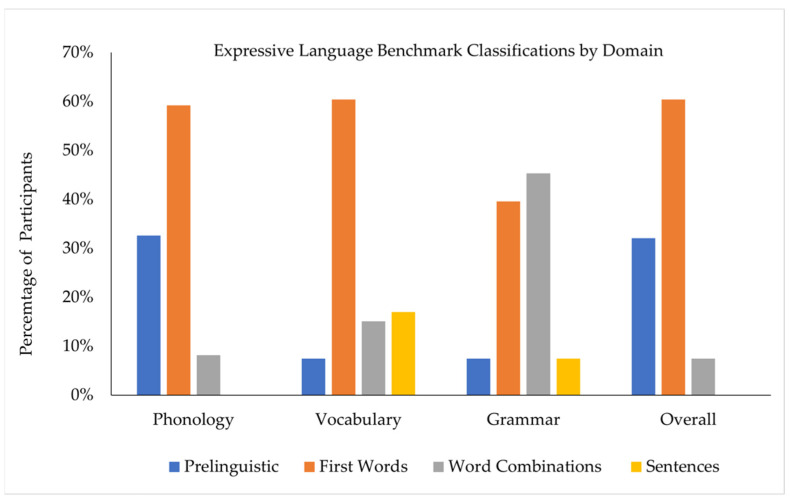
Expressive Language Benchmarks as a Function of Language Domain. Note. Phonology domain sample size = 49 (due to incomplete GFTA-3 administrations or examiner error). Overall ELB classifications were not impacted by the missing GFTA-3 scores.

As seen in Figure 1, variability was observed in the number of children assigned to each benchmark across the three language domains. In both the phonology and vocabulary domains, the largest proportion of children was classified in the first words ELB. In the grammar domain, the largest proportion of children was classified in the word combinations ELB. In both the phonology and grammar domains, the smallest proportion of children was classified in the sentences ELB; in the vocabulary domain, the smallest proportion of children was classified in the preverbal ELB.

Descriptive statistics were also computed to characterize the chronological ages of the children assigned to each ELB across language domains. Although, in general, mean chronological age increased as ELBs increased, considerable variability was observed in the chronological ages of the children assigned to the different ELBs (Table 3).

As noted previously, the PLS-5, which was used to define the grammar ELB, does not exclusively assess grammar skills. Therefore, children can potentially earn an age-equivalent score that would assign them to a grammar ELB but not demonstrate the skills suggested by the “name” of that benchmark (e.g., preverbal, first words, word combinations, or sentences). Follow-up analyses were conducted to determine whether any child who was assigned to a grammar ELB demonstrated grammatical benchmarks that were different from what was assumed by their assigned ELB level (e.g., grammar ELB assigned via the PLS-5 age-equivalent score was first words, but no labels were observed for the child on the PLS-5; or the assigned grammar ELB was word combinations, but 4–5-word sentences were observed on the PLS-5). Results indicated that 34% of children in the present sample were assigned to a grammar ELB that was not consistent with the skills observed during their assessment. More specifically, 20.8% of children were assigned to a grammar ELB but did not yet demonstrate that specific benchmark (i.e., six children in first words ELB were not yet able to produce a label, and five children in the word combinations ELB were not yet able to produce word combinations on the PLS-5), and 13.2% were assigned to a grammar ELB but were observed to produce the grammatical skill indicative of the next higher benchmark (i.e., three children in the first words ELB who produced word combinations and four children in the word combinations ELB who produced 4–5-word sentences).

**Figure 2:**
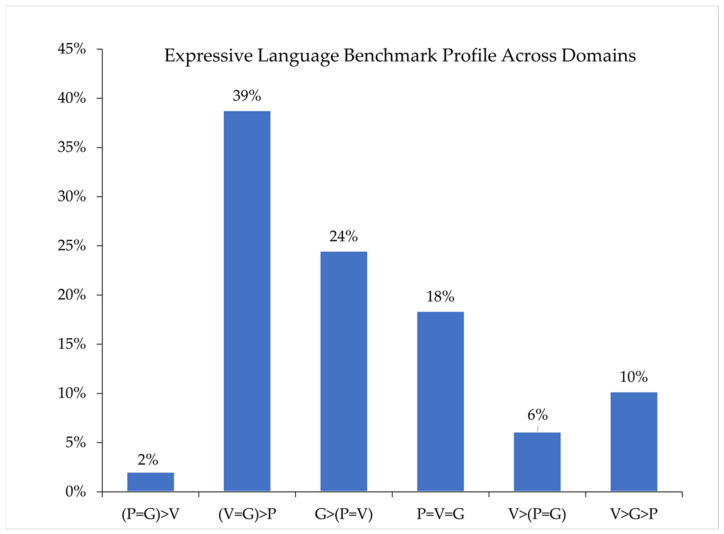
Expressive Language Benchmarks Profiles Across Language Domains (*n* = 49). Note. P, phonology domain; V, vocabulary domain; G, Grammar domain; “=”, same benchmark; “>”, higher benchmark.

Next, we considered children’s ELB profiles across language domains (Note: the four participants with incomplete phonology data were excluded from this analysis). Overall, six patterns of ELBs across language domains were observed (see Figure 2). Approximately 18% of participants achieved the same ELB across all three language domains; 72% of participants achieved the same ELB in two-thirds of language domains, and 10% of participants achieved a different ELB in each language domain.

Finally, despite the variability observed in ELBs across language domains, significant associations were observed between all pairs of ELB domains. The Spearman rank correlation between phonology ELB and vocabulary ELB (rank order of ELB) was *r*_s_ = 0.54, *p* < 0.001; the Spearman correlation between phonology ELB and grammar ELB was *r*_s_ = 0.77, *p* < 0.001; and the Spearman correlation between vocabulary ELB and grammar ELB was *r*_s_ = 0.64, *p* < 0.001.

### 3.2. Correlates of Expressive Language Benchmarks

We also considered the association between ELBs and other measures of participant performance (see Table 4). First, using Spearman rank correlations, we considered the associations between the ELBs and the language measures not included in the ELB criteria. Significant positive associations were observed across all comparisons.

In addition, the association between the ELBs and other developmental characteristics known to relate to language ability, specifically chronological age, nonverbal cognitive ability, and verbal short-term memory were considered (see Table 5). Significant positive associations were observed across all comparisons.

### 3.3. Longitudinal Changes in Expressive Language Benchmarks

Finally, changes in ELBs were considered for the 14 children in the present study for whom we had longitudinal follow-up data. Overall, changes in ELBs were observed for most of the children, with 28.6% of children demonstrating ELB changes in one domain, 35.7% of children in two domains, and 21.4% of children in three domains. In Table 6, we present descriptions of the changes observed as a function of language domain. Moreover, Wilcoxon signed-rank tests indicated significant changes in vocabulary (*z* = 2.60, *p* = 0.009, *r* = 0.70) and grammar ELBs (*z* = 2.45, *p* = 0.014, *r* = 0.66). The comparisons considering changes in phonology (*z* = 1.90, *p* = 0.058, *r* = 0.60) and overall ELBs just failed to reach criterion for significance (*z* = 1.90, *p* = 0.058, *r* = 0.51).

## 4. Discussion

Characterizations of behavioral phenotypes, descriptions of developmental trajectories, and decisions regarding treatment efficacy all rely on outcome measures [5,36,41]. To this end, an NIDCD-formed panel of experts outlined a framework for defining ELBs to facilitate comparisons of language progress across different assessment measures [5]. This approach to describing development and individual differences in development has the potential to facilitate comparisons across studies that use very different measures of language ability, thereby allowing for a fuller and more accurate characterization of the DS phenotype. This enhanced understanding of the phenotype is essential for exploring the neurological bases of language and cognition in DS. Language is a high-priority intervention target in children with DS, and early intervention can prevent deceleration in language growth and create a cascade of positive events that can have cumulative, pervasive, and long-lasting benefits [36]. Thus, there is a critical need to identify methods of supporting descriptive and treatment research that facilitates our understanding and support of language development during the transition from prelinguistic communication to the production of sentences [36]. The language phenotypes of children with DS differ in important ways from children with autism, and thus, it is necessary to evaluate the utility of the ELB framework for characterizing children with DS.

In the present study, we used the ELB procedures to characterize the expressive language skills of children with DS between 2.50 and 7.99 years of age. Using this approach, we documented considerable variability in language performance, with the sample distributed across the range of ELBs and intra-individual variability in ELB assignments and in the chronological ages of children observed within ELB assignments across language domains. In both the phonology (56%) and vocabulary (64%) domains, most children were assigned to the first words ELB. In the grammar domain, 45% of children (the largest proportion) were assigned to the word combinations ELB. We also found that only 15% of the children in the sample had the same ELB across language domains. Phonological skills were an area of relative challenge, with 49% of children demonstrating a phonological ELB lower than their ELBs in both vocabulary and grammar. Comparisons of performance across the vocabulary and grammar domains indicated that 55% of our sample demonstrated the same ELB in both domains, 18% demonstrated vocabulary skills that were stronger than grammar skills, and 27% demonstrated grammar skills that were stronger than vocabulary skills.

Some of the patterns observed are consistent with prior research on the DS language phenotype when also considering the ELB categories’ reduced specificity. For example, Karmiloff-Smith et al. [42] called investigators to move beyond treating individuals with DS as a homogenous group and recognize the considerable heterogeneity observed among individuals with DS across domains. Indeed, the ELB procedures highlight considerable variability among individuals with DS both within and across language domains. Moreover, the influences of cognitive limitations, hearing loss, and other anatomical and physiological features of the DS phenotype contribute to phonological challenges [10]. Here again, our findings using the ELB procedures are consistent with this pattern, with ELBs in the phonology domains lower than at least one of the other domains in approximately 80% of the children assessed and at the same as the overall ELB (defined by the lowest benchmark observed across domains) for all but one child.

At the same time, patterns observed do not map perfectly onto expectations based on prior research. For example, considering group-level performance, previous research indicates that vocabulary skills are an area of strength relative to grammar skills in individuals with DS [2]. Although approximately 70% of children in our sample demonstrated vocabulary skills at the same ELB or a higher ELB than grammar skills, only 16% demonstrated the specific profile in which the vocabulary ELB was higher than the grammar ELB. This difference is due to the fact that benchmarks, by their very nature, are less precise than the data generated by detailed investigations of language development. It is likely that when used in groups that demonstrate more uniform and predictable language profiles, such as children with TD, the precision of the ELB profiles would be significantly better than when used with groups, such as children with DS, who demonstrate more variable trajectories across different language domains.

Additional research characterizing the language trajectories across domains, paired with the ELB approach, would clarify if and how benchmark definitions map onto detailed characterizations of language trajectories and inform our understanding of the utility of ELBs when characterizing language profiles in children with DS or other disability groups. Specifically, more precise characterizations of the association between vocabulary and grammatical skills in children with DS and how these patterns align with performance on tests such as the PLS-5 could help make adjustments to the minimum criteria so that the ELBs align more closely with what is known about the DS language profile. For example, it may be that because of the slower rate of language development observed in children with DS, using the scores corresponding to the 25th percentile rather than the 50th percentile for the target developmental age would improve the alignment of the ELBs across domains.

The results also highlight the limitations of many available standardized tests within an ELB framework. For example, because the PLS-5 summarizes performance across multiple language domains (e.g., vocabulary, pragmatics, grammar), when using this tool as a proxy for grammar, it is important to recognize that the ELB assignments may not fully align with observed grammar milestones. In the present study, approximately 34% of children were assigned to a grammar ELB using their PLS-5 scores that did not match the grammar milestones observed during the assessment. Importantly, Tager-Flusberg et al. [5] strongly recommended considering performance from multiple sources (e.g., direct standardized assessments, naturalistic language samples, parent-report measures) to obtain a representative sample of child communication skills, and the present findings reinforce that recommendation. In particular, the grammar domain may be better assessed using naturalistic sampling methods or other techniques that would allow the criterion to be based more tightly on grammatical skill levels.

Tager-Flusberg et al. [5] acknowledged multiple challenges to applying the ELB framework. First, few assessments that are currently available consider developmental performance across the full ELB range, and many yield scores that are not pure measures of any single language domain. In addition, as a starting point, the ELB procedures were based on estimates of when different language milestones are observed in children with TD [5]. Age-equivalent scores corresponding to these developmental estimates are then used to derive the final ELBs. Notably, Tager-Flusberg et al. [5] cautioned that these age-equivalent ranges are rough estimates at best. Age-equivalent scores reflect the median level of performance for children at that designated age [43]. Thus, age-equivalent scores provide an imprecise estimate of developmental level. Future research focused on elucidating the timing of expressive language milestones relative to performance on standardized test items, both in children with neurodevelopmental disabilities and in children with TD, would be helpful for understanding and potentially refining the ELB criteria.

Concerning the assignment of children to an overall ELB, the majority of the children in our sample were assigned to the first words ELB (59%). Because the overall ELB requires children to meet the minimum criteria in all domains, it is highly influenced by areas of challenge in children who demonstrate variability in their language profiles across domains. Thus, similar to recommendations made for standardized assessment scores, the overall ELB will not be a valid representation of a child’s skill level when variability across domains is observed. For example, of the children who earned at least one ELB at the word combinations or sentence stage, nearly all were classified at a lower overall ELB because their phonology ELBs were lower (16/17 for vocabulary and 26/28 for grammar).

Despite the variability observed across language domains, ELB rankings were significantly correlated across language domains, with language validation measures not included in the ELB assignments and with measures known to be associated with language performance. ELB rankings were also significantly associated with participant chronological age, nonverbal cognitive ability, and verbal short-term memory. Together, these data suggest that the ELB rankings are useful in capturing progress in language provided that the cautions about intra-individual variability are kept in mind.

Finally, we considered changes in ELBs for a subset of participants who had completed their follow-up visits 18–24 months after the initial session. ELB changes were observed in 86% of these participants. Moreover, significant improvement in ELB classification were observed in both the vocabulary and grammar domains, supporting the utility of the ELB approach for characterizing, in broad strokes, progress in language development. The ELB rankings for the phonology domain did not meet the criterion for a significant change, nor did the overall ELB. These patterns, however, likely would be significant with a larger sample size given the effect sizes observed (phonology: *r* = 0.60, overall: *r* = 0.51). Additional research focused on elucidating the rate of growth in children with DS across a wide range of ages will facilitate our understanding of the utility of ELBs.

## 5. Limitations

Findings from the present study provide an important first step in considering the utility of the ELB procedures for use in young children with DS. Nonetheless, there are some limitations worth noting. Specifically, the present study only utilized standardized assessment performance when assigning ELBs. Additional research considering the utility of naturalistic language sampling in assigning ELBs and the value of combining different assessment methods when defining ELBs is needed. In addition, it remains unknown the extent to which ELB assignments are impacted by the assessments used in the classification process. In addition, studies that combine in-depth language phenotyping with ELB classification procedures are needed to elucidate the sensitivity of the ELBs in characterizing the DS language phenotype over time and the extent to which the ELBs are sensitive to change over time. Indeed, in the present study, longitudinal data were only available for a small group of children. Larger longitudinal datasets are needed to clarify the extent to which ELBs are sensitive to changes over time and if these changes are impacted by participant characteristics (e.g., cognitive level, initial ELB, etc.).

## 6. Conclusions

In sum, the application of ELBs, as outlined by Tager-Flusberg et al. [5], has both strengths and limitations. The ELBs have the potential to be a useful tool for both characterizing the DS behavioral phenotype and monitoring changes in language performance as individuals transition from the preverbal to sentence stages of expressive language. Using ELBs, we documented considerable heterogeneity among children with DS. Across all ELBs, significant associations were observed with language measures not used in the classification process and with performance in other developmental domains known to be associated with language development. It is important to note that, in populations that demonstrate variability in their language profiles across domains, the overall ELB may be only an imperfect representation of a child’s skill level. In addition, more incongruities may be observed when comparing ELB profiles to more detailed characterizations of language profiles although more research is needed to validate this hypothesis Finally, limitations in currently available standardized assessment measures and variability across assessment methods should be considered when used to assign ELBs (e.g., range of scores considered, domains assessed). Our data also reinforce Tager-Flusberg et al.’s recommendation to consider performance across multiple sources to obtain a representative sample of a child’s communication skills. Future research considering the timing of expressive language milestones relative to children’s language performance on language assessments across domains would be helpful for understanding and potentially refining the ELB criteria.

## Figures and Tables

**Table 1:** Expressive Language Benchmarks Minimum Criteria as a Function of Language Domain.

Benchmark	Phonology	Vocabulary	Grammar
Preverbal (<15-month AE)	Does not meet the minimum criterion for First Words ELB		
First Words (15-month AE)	Screener: 4 consonants	CDI-W&G: 20 words	PLS-5: Raw score = 20
Word Combinations (24-month AE)	GFTA-3 SiW: Raw score = 68 (for boys and girls)	CDI-W&S: 297 words	PLS-5: Raw score = 28
Sentences (36-month AE)	GFTA SiW: Raw score = 35 for girls or 39 for boys	DAS-II Naming Vocabulary: Ability score = 91	PLS-5: Raw score = 36

Note. AE, age-equivalent score; Screener, Articulation Screening Test; GFTA-3 SiW, Goldman-Fristoe-Test of Articulation-3 Sounds in Words, CDI-W&G, MacArthur–Bates Communicative Development Inventory Words and Gestures; CDI-W&S, MacArthur–Bates Communicative Development Inventory Words and Sentences, DAS-II, Differential Ability Scales-II; PLS-5, Preschool Language Scale-5.

**Table 2:** Descriptive statistics for the language measures used to define ELBs.

Measures	M (SD, Range)
Articulation Screening Phonology Score (number of consonants out of 8)	5.17 (2.48, 0–8)
GFTA-3 SiW Raw Score ^a^	79.79 (35.32, 3–128)
CDI-W&G: Expressive Vocabulary Size (in words)	155.33 (117.74, 4–381)
CDI-W&S: Expressive Vocabulary Size (in words)	211.65 (188.94, 4–635)
DAS-II Naming Vocabulary Ability Score	54.42 (34.10, 10–116)
PLS-5 EC Growth Score	27.45 (5.73, 15–38)

^a^*n* = 33. Note. GFTA-3 SiW, Goldman–Fristoe Test of Articulation-3 Sounds in Words, CDI-W&G, MacArthur-Bates Communicative Development Inventory Words and Gestures; CDI-W&S, MacArthur-Bates Communicative Development Inventory Words and Sentences; DAS-II, Differential Ability Scales-II; PLS-5 EC, Preschool Language Scale-5 Expressive Communication.

**Table 3:** Descriptive Statistics for the Chronological Ages (in Years) of Children Assigned to the Expressive Language Benchmarks as a Function of Language Domain.

Benchmark	Phonology M (SD, Range)	Vocabulary M (SD, Range)	Grammar M (SD, Range)	Overall M (SD, Range)
Preverbal	4.09	3.32	4.13	4.05
	(1.44, 2.57–7.98)	(0.44, 2.67–3.65)	(0.98, 3.47–5.58)	(1.40, 2.57–7.98)
First Words	5.08	4.58	4.40	5.01
	(1.47, 2.92–7.86)	(1.47, 2.57–7.98)	(1.60, 2.57–7.98)	(1.44, 2.73–7.86)
Word Combinations	6.00	4.52	5.06	6.00
	(1.36, 5.02–7.98)	(0.97, 3.38–6.14)	(1.45, 2.73–7.98)	(1.36, 5.02–7.98)
Sentences	N/A	6.33	5.74	N/A
		(1.21, 4.41–7.98)	(1.33, 4.41–7.54)	

Note. M, mean; SD, standard deviation.

**Table 4:** Spearman Rank Correlations between Expressive Language Benchmarks and Language Validation Measures.

Language Validation Measure	Phonology ELB	Vocabulary ELB	Grammar ELB	Overall ELB
DAS-II Verbal Comprehension	0.65 **	0.62 **	0.73 **	0.67 **
CDI-W&S Grammar Complexity	0.57 **	0.67 **	0.56 **	0.58 **
Vineland-3 Receptive Communication	0.41 **	0.56 **	0.41 **	0.43 **
Vineland-3 Expressive Communication	0.72 **	0.72 **	0.72 **	0.71 **

** *p* < 0.001. Note. ELB, Expressive Language Benchmark; DAS-II, Differential Ability Scales; CDI-W&S, MacArthur-Bates Communicative Development Inventory Words and Sentences.

**Table 5:** Spearman correlation coefficients between Expressive Language Benchmarks and Broader Developmental Characteristics.

Developmental Characteristic	Phonology ELB	Vocabulary ELB	Grammar ELB	Overall ELB
Chronological Age	0.41 *	0.45 **	0.32 *	0.40 *
DAS-II Picture Similarities	0.46 **	0.56 **	0.50 **	0.49 **
DAS-II Recall of Digits—Forward	0.54 **	0.66 **	0.65 **	0.51 **

* *p* < 0.05, ** *p* < 0.001. Note. ELB, Expressive Language Benchmark; DAS-II, Differential Ability Scales-II.

**Table 6:** Longitudinal Changes in Expressive Language Benchmarks (*n* = 14).

Benchmark	Phonology ELB ^a^	Vocabulary ELB	Grammar ELB	Overall ELB
Decreased 1 ELB	1	0	0	1
No Change	3	6	8	7
Increased 1 ELB	5	5	6	5
Increased 2 ELBs	1	3	0	1

Note. ELB, Expressive Language Benchmark. ^a^ Data missing for four participants. Three participants could not complete the GFTA-3 at initial visit; for one participant, the recording failed to save at follow-up visit.

## Data Availability

The datasets used and/or analyzed for the present paper can be made available upon a reasonable request to the corresponding author.
